# Does green credit enhance corporate green total factor productivity?

**DOI:** 10.3389/fpubh.2025.1574787

**Published:** 2025-03-28

**Authors:** Bo Zhang, Jiayan Dong, Meiyi Xiong, Xiang Gao

**Affiliations:** ^1^School of Economics and Management, Hefei University, Hefei, China; ^2^Research Center of Finance, Shanghai Business School, Shanghai, China

**Keywords:** green credit, green total factor productivity, green innovation, green transformation, high-quality economic development, sustainability

## Abstract

Green total factor productivity (GTFP) constitutes a fundamental driver for corporate green transitions, closely related to the United Nations sustainable development goals and what China pursues for “new quality productive forces.” As a result, it becomes crucial to find out if and how government green credit policies can improve corporate GTFP. To answer these questions, this study uses the Data Envelopment Analysis (DEA) Epsilon-Based Measure (EBM) model and the Malmquist index to create a novel firm-specific GTFP proxy. We find that macro green credit positively and statistically significantly affects corporate GTFP. Further analysis reveals that the concerned effect is functionary through two channels—R&D investment and public supervision. The effect is more prominent for companies in the growth stage, going through a digital transformation, and working in non-polluting or heavily polluting industries. The implications for a sustainable economy are multifaceted from the perspective of policymakers, such as formulating a series of green credit policies on a local level, facilitating environmental information access, and getting every market participant involved and motivated.

## 1 Introduction

A key way to achieve high-quality development, create long-lasting low-carbon economies, and boost new quality productivity is for corporations to increase their green total factor productivity. Despite this, businesses do not do much to improve GTFP because the green transition requires a lot of money, takes a long time to pay for itself, and returns are hard to predict (1). The Green Credit Guidelines (GCG), introduced by the former China Banking Regulatory Commission, now serve as an important policy instrument for steering corporate green transformation. This regulatory framework establishes standardized implementation pathways for green financial interventions (2). The Central Financial Work Conference in 2023 clearly highlights the important role of green finance in promoting sustainable development, positioning it as both a cornerstone of China’s high-quality economic growth and a strategic enabler of “New Quality Productivity Forces.” And new quality productivity forces not only focus on the improvement of production efficiency, but also emphasizes the greening and sustainability of the production process, which is the core power to promote high-quality economic development. Enhancing corporate GTFP rests critically with robust financial capital backing, with green credit emerging as a central mechanism. Therefore, looking into how green credit boosts this productivity and judging the results of its policies has huge implications for coordinating financial plans with environmental goals. This analysis not only validates the efficacy of existing frameworks but also informs future innovations in green finance.

At present, the financial instruments aimed at enhancing corporate GTFP predominantly revolve around green credit, green bond policies, and carbon emission reduction policies. First, green credit and green bonds help companies become more environmentally friendly by making better use of capital and increasing access to long-term financing. This speeds up technological and environmental progress (3, 4). Carbon emission reduction policies encourage companies to use less energy, improve ESG (environmental, social, and governance) practices, and make sustainability reporting clearer. All these things lead to growth in GTFP (5). Many studies that have already been done (6) focus on how green financial policies at the macro level affect the green behavior of companies at the micro level. However, can green financial policies at a macro level effectively address the heterogeneous green innovation needs of diverse enterprises? Does the enforcement of green credit inadvertently heighten financial barriers for firms, resulting in insufficient capital investment, which in turn weakens the motivation of enterprises to improve GTFP? Institutional frameworks for green credit and innovation are getting better, but it’s still not clear how these policies fit in with how businesses operate. How does green credit impact corporate GTFP in terms of causality? Through what mechanistic pathways does green credit influence GTFP? This study aims to fill in the gaps between theory and practice by shedding light on these issues. It will then provide useful information on how to make the most of green finance tools to promote corporate sustainability while minimizing the negative effects of unintended policy choices.

In contrast, from the perspective of short-term effects of policy implementation and behaviors of financial institutions, the paper emphasizes the problems of information asymmetry or credit crunch that may occur in the process of policy implementation. This research employs panel data from A-share listed companies (2008–2021) to investigate how green credit influences corporate GTFP. Its key innovations are threefold. Firstly, we use the DEA-EBM model and the Malmquist index to measure GTFP while keeping economic performance and environmental outcomes in balance. This gives a full picture of how green credit helps businesses grow in a way that is both profitable and good for the environment. Second, this paper focuses on the importance of public oversight as a key outside force in putting green credits into action. It reveals how public scrutiny works with corporate governance to make policies more effective. The findings are that regulatory frameworks and stakeholder-driven accountability can work together to make policies more effective. Third, the study finds different effects of green credit by evaluating at different levels of digital transformation and stages of an enterprise’s life cycle. Such differences can help policymakers come up with more effective solutions.

## 2 Theoretical framework and hypothesis development

### 2.1 Green credit and green total factor productivity

Improving corporate GTFP is a catalyst for high-quality economic development. Part of the reason businesses are encouraged to go green is so they can achieve sustainable development. Another reason is so they can achieve green and high-quality development in line with national policy guidance (7). Also, because increasing GTFP during the green transition requires a lot of money and “positive externality,” managers who believe in the “rational man” will not take the initiative to work on green transformation projects. This is why policy guidance is needed to help them decide how to spend their money. Green credit, on the other hand, can encourage businesses to actively engage in green production activities, take responsibility for the negative external effects of social production activities, and then adjust the opportunity cost of businesses going green on the fly. At the same time, to obtain favorable loan return (8) and additional competitiveness (9), enterprises gradually switch to green production, thus promoting corporate GTFP.

According to the theory of externalities, green credit can require banks to carefully examine polluting companies and projects before giving them credit. They should also “make explicit” the hidden environmental costs and “internalize” the external environmental costs (10). For example, the pollution that businesses cause should be reflected in the cost of borrowing money, which would reduce the difference between the private and social costs for those businesses and boost the environmental governance of their own operations (11). Therefore, companies will proactively channel funds into green initiatives to reduce pollution projects and improve GTFP. Furthermore, the theory of Porter’s hypothesis predicts that green credit will have a positive impact on corporate green innovation (12, 13). Green credit reduces the uncertainty tied to corporate green investment; not only can it ensure that enterprise green innovation can play a role, but it can also broaden enterprise financing channels and support enterprise research and development innovation. By incentivizing corporate innovation, green credit can enhance resource allocation and production efficiency, thus improving enterprise GTFP. Grounded in the preceding theory and literature analysis, the paper posits the first research hypothesis.

*H1:* Green credit is beneficial to improve the green total factor productivity of enterprises.

### 2.2 The mechanism of green credit enhancing firm green total factor productivity

By supplying green funds, green credit can strongly motivate firms to boost R&D investment, thereby significantly enhancing their R&D innovation willingness (11). When enterprises increase R&D investment and leverage the funds toward green innovation, their corporate GTFP will improve through the following avenues. The first avenue is the research and development of pollution emission filtration. Cleaning and recycling can directly reduce the pollution in the production process of enterprises, improve the ability of enterprises to control contamination, and thereby enhance corporate GTFP. Second, businesses can improve the production process and get rid of old production capacity by researching and developing advanced production technology and resource utilization efficiency. This makes green production more efficient. Furthermore, the public, being highly sensitive to corporate pollution, plays a crucial role in implementing the policy. Green credit improves China’s environmental protection policy system and feedback mechanism for environmental protection issues. It also makes people more aware of environmental issues and acts as an outside watchdog for businesses. People who watch over businesses can change the way corporate managers act by using public opinion to stop them from avoiding their environmental responsibilities for their own benefit (14). As a main part of the environment, companies that are supervised from the outside will improve their production process through rationalization measures, make better use of resources, and make better investment decisions with a positive mindset. This will lower the pollution that companies release into the environment. This is especially the case when the degree of environmental pollution is relatively serious. Public oversight can have a bigger effect on management’s decisions, making green resource use much more efficient and helping to raise corporate GTFP (15) by acting as a voice for environmental protection. Based on the above analyses, we hence put forward the following set of hypotheses:

*H2a:* Green credit can increase the investment in R&D and innovation of enterprises, thus improving GTFP.

*H2b:* Green credit can enhance the public's supervision on the environment, urge enterprises to reduce pollution, and then improve GTFP.

## 3 Empirical strategy

### 3.1 Estimation specification

To explore how green credit affects corporate GTFP, this study employs a dual fixed effect model for empirical analysis. The paper formulates the estimation (Equation 1) as follows:

(1) $$GTFP_{\mathrm{it}}=\alpha_{0}+\alpha_{1}GCRE_{\mathrm{it}}+\beta X_{\mathrm{it}}+\lambda_{i}+\mu_{t}+\in_{\mathrm{it}}\text{,}$$

where $GTFP_{\mathrm{it}}$ represents the green total factor productivity of the i enterprise in the t period, i.e., the GTFP of firm i during year t. $GCRE_{\mathrm{it}}$ represents the intensity of green credit in the region where the firm i is headquartered for the t period. $X_{\mathrm{it}}$ represents the vector of control variables. $\lambda_{i}$ and $\mu_{t}$ represent firm and time fixed effect, respectively, and $\in_{\mathrm{it}}$ is the usual error term.

As for the estimated coefficients, $\alpha_{0}$ represents constant term, $\alpha_{1}$ represents the regression coefficient of GTFP against the green credit. If $\alpha_{1}$ turns out to be positive, it suggests that green credit has a positive influence on corporate GTFP; If $\alpha_{1}$ is otherwise negative, it implies that green credit adversely affects corporate GTFP. β represents the regression coefficient vector associated with a list of control variables.

### 3.2 Variable construction

#### 3.2.1 Dependent variable

GTFP is the dependent variable that we are studying its potential determinants. To use the DEA method suggested by Sun et al. (8), we choose labor, capital, and intermediates for raw materials and energy as the variables that are being studied. The labor input is assessed by the number of employees, the capital input is measured by the net fixed assets, and the intermediate input is determined by the cash paid for goods and services. Operating income measures the expected output. Meanwhile, corporate pollution emissions, including industrial wastewater and industrial waste gas, are considered undesired outputs. The calculation of pollution value is based on the methodology of Zhou et al. (16). The amount of chemical oxygen demand and ammonia nitrogen that leave businesses is used to measure their wastewater discharge. Similarly, the amount of sulfur dioxide (SO₂) and nitrogen dioxide (NO₂) that leave businesses is used to measure their exhaust gas discharge. We standardize the pollution equivalent values, originally sourced from the document “Administrative Measures for the Collection of Pollution Emission Charges.”

#### 3.2.2 Explanatory variable

The concerned explanatory variable in this study is Green Credit (GCRE). The impact intensity of green credit received by firms is measured using the inverse index of interest expenses for the six energy-intensive industries in the province where the firms are located.

#### 3.2.3 Controls

Drawing on the work of Zhang et al. (17) for reference, this study selects control variables at the enterprise level and macro level as follows: (1) Firm SIZE. This represents the total scale of an enterprise, measured by the logarithm of its total assets. (2) Asset-liability ratio (LEV). The ratio of total liabilities to total assets measures the enterprise’s ability to pay its debts and conduct its business with the funds of creditors. (3) Return on Assets (ROA). The ratio of net profit to total assets measures the enterprise’s profitability and asset utilization efficiency. (4) Fixed assets turnover rate (TAR). It reflects the enterprise’s operational ability and management level, influencing its production and operation status. The ratio of business income to the average net fixed assets is used to measure it. (5) SHARE concentration. This will have an impact on corporate governance, as measured by the aggregate shareholding of the top ten largest shareholders. (6) Gross domestic product (GDP). It reflects the economic development of the region where the enterprise is located and affects the regional industrial structure to a certain extent. The logarithm of the provincial GDP in the enterprise’s location serves as its measurement. (7) The level of foreign investment (FDI) is also considered. This reflects the degree of economic openness in the region and influences the development of local financial markets. We measure it using the logarithm of the total foreign investment in the province where the enterprise is located.

### 3.3 Data sources and the sample

This study looks at how green credit affects corporate GTFP by using panel data from A-share companies that traded on the Shanghai and Shenzhen stock exchanges from 2008 to 2021. Using relevant literature as a guide, we implement the following processing steps to ensure data reliability: (1) getting rid of companies that do not have enough data; (2) leaving out companies that do not have any observation years; and (3) applying a double-sided 1% tail reduction to enterprise-level continuous variables to lessen the effects of extreme value effects. The data are taken from a variety of sources, including the CSMAR Database, CNRDS Database, Annual Report of Listed Companies, China Industrial Statistics Yearbook, and China Statistical Yearbook on Environment. After the above data processing steps, we are left with 1,244 publicly listed firms with a total sample size of 17,416 after considering time series data. Table 1 provides the descriptive statistics for all variables.

**Table 1:** Descriptive statistics.

Variable	Sample size	Average value	Standard deviation	Minimum value	Maximum
GTFP	17,416	1.124	0.594	0.318	4.740
GCRE	17,416	0.536	0.137	0.094	0.972
SIZE	17,416	13.215	1.419	9.872	16.999
LEV	17,416	0.518	0.210	0.075	1.096
ROA	17,416	0.052	0.072	−0.233	0.283
TAR	17,416	8.768	20.451	0.224	147.784
SHARE	17,416	0.532	0.154	0.209	0.889
GDP	17,416	10.938	0.554	9.085	12.123
FDI	17,416	7.381	1.451	1.675	10.720

The GTFP statistics reveal significant heterogeneity among our sample firms, with a mean of 1.124, a standard deviation of 0.594, and a range spanning from 0.318 to 4.740. These disparities may stem from divergent operational practices or varying corporate prioritization of environmental sustainability. The standard deviation of firm size and fixed assets turnover rate is high for micro-level controls. This means that there are big differences between listed companies when it comes to their total assets, operating capacity, and management level. At the same time, this may also be due to different industries in which different enterprises are located and different production and operation modes in different industries. When looking at macro-control variables, foreign direct investment (FDI) varies a lot from province to province (SD = 1.451), which shows noticeable differences in how open and developed financial markets and economies are in different areas.

## 4 Empirical results and analysis

### 4.1 Benchmark regression

Table 2 presents benchmark regression results across three specifications. Column (1) only adds the green credit index and does not include any control variables. Columns (2) and (3) progressively incorporate enterprise-level and macro-level controls. The GCRE coefficient stays positive and statistically significant at the 1% level across all models. This proves that green credit significantly increases corporate GTFP, which means that Hypothesis 1 is true. Combined with the actual development, green credit takes environmental factors into consideration in corporate credit and strengthens the strict examination and approval of pollution projects. By internalizing environmental costs, green credit policies compel firms to reduce investments in polluting activities and transition toward sustainable projects. At the same time, green credit guides businesses in a smart way toward green innovation while also making the use of funds and resources more efficient and encouraging businesses to change and improve through green innovation. In addition, green credit can also ease the information asymmetry, thereby enhancing corporate GTFP.

**Table 2:** Benchmark regression results.

Variable	(1)	(2)	(3)
	GTFP	GTFP	GTFP
GCRE	0.345*** (3.56)	0.312*** (3.30)	0.317*** (3.35)
SIZE		0.143*** (8.01)	0.142*** (8.00)
LEV		−0.167*** (−3.00)	−0.166*** (−2.97)
ROA		0.659*** (8.08)	0.658*** (8.08)
TAR		0.010*** (9.94)	0.010*** (9.95)
SHARE		0.087 (1.10)	0.090 (1.16)
GDP			0.072 (1.48)
FDI			−0.010 (−0.71)
Constant term	0.784*** (16.75)	−1.019*** (−4.61)	−1.690*** (−3.17)
Sample size	17,416	17,416	17,416
R2	0.124	0.313	0.314
Id effect	Yes	Yes	Yes
Year effect	Yes	Yes	Yes

### 4.2 Robustness tests

#### 4.2.1 Using alternative measures

The baseline specification applies the super-EBM model to figure out how efficient the business is, and then the Malmquist index is multiplied to find the GTFP growth rates for each period. The first alternative is to directly measure enterprise efficiency without multiplying the Malmquist index like in Qi et al. (18). The second alternative involves measuring the enterprise efficiency value by a globally referenced non-oriented super-efficiency SBM model that incorporates variable returns to scale and unexpected outputs. Columns (1) and (2) in Table 3 show that our concerned green credit coefficient remains statistically significant when alternative GTFP proxies are used.

**Table 3:** Regression results after substitution of variables and reduction of sample interval.

	(1)	(2)	(3)	(4)
	GTFP_MAL	GTFP_SBM	GTFP	GTFP
GCRE	0.167*** (5.51)	0.277*** (2.74)	0.265*** (2.99)	0.234*** (3.01)
SIZE	−0.007** (−1.97)	0.165*** (8.49)	0.119*** (6.02)	0.124*** (5.81)
LEV	0.108*** (6.39)	−0.039 (−0.65)	−0.160** (−2.50)	−0.156** (−2.32)
ROA	0.589*** (14.90)	1.384*** (12.69)	0.539*** (6.46)	0.479*** (5.88)
TAR	0.004*** (19.82)	0.011*** (12.21)	0.010*** (10.40)	0.009*** (11.04)
SHARE	0.074*** (3.34)	0.274*** (3.47)	0.029 (0.35)	−0.031 (−0.35)
GDP	0.029** (2.16)	0.078 (1.04)	0.180*** (2.92)	0.142** (2.31)
FDI	−0.007 (−1.40)	0.070 (1.16)	−0.015 (−1.17)	−0.015 (−1.28)
Intercept	0.557*** (3.83)	0.014 (0.83)	−2.527*** (−3.81)	−2.128*** (−3.13)
Sample size	17,416	17,416	12,440	9,952
R2	0.143	0.283	0.297	0.302
Id effect	Yes	Yes	Yes	Yes
Year effect	Yes	Yes	Yes	Yes

^**,***^Represent significance level at 5 and 1%, respectively.

#### 4.2.2 Narrowing the sample interval

This research employs panel data from Shanghai and Shenzhen A-share listed firms over the period from 2008 to 2021 to assess green credit policy effects. To avoid the statistical deviation caused by the particularity of sample time range selection, the sample time range is changed and regressed. The China Banking Regulatory Commission’s promulgation of the Green Credit Guidelines in January 2012 established a standardized framework for green financing practices; in June 2014, the former CBRC introduced “Key Evaluation Indicators for Green Credit Implementation,” which became a key document for green credit evaluation; these important policy documents have continuously improved China’s green credit and gradually enhanced the implementation of the policies. To mitigate single-policy bias, two distinct sample periods are selected as 2012–2021 and 2014–2021, with outcomes reported in columns (3) and (4). And the green credit coefficient remains statistically significant across both periods, confirming result stability despite temporal adjustments.

#### 4.2.3 Changing fixed effect dimensions

If one only looks at enterprise-level fixed effects, it is possible to encounter statistical bias because they do not consider changes in GTFP (19). Building on Wang and Zhang's (20) work, this study includes fixed effects at the industry and province levels. Table 4 shows the full regression results. In Column (1), time and industry fixed effects are included, and the standard errors are clustered at the industry level. In Column (2), time and province fixed effects are incorporated, with cluster-robust standard errors applied at the province level. In Column 3, the time fixed effects are added to the industry and province fixed effects, and the cluster-robust standard errors are found at the industry-province level. Table 4 demonstrates that the green credit coefficient remains significantly positive across alternative fixed effect specifications.

**Table 4:** Changing fixed effect dimensions.

Variable	(1)	(2)	(3)
	GTFP	GTFP	GTFP
GCRE	0.278*** (3.14)	0.318** (2.37)	0.312*** (2.60)
SIZE	0.110*** (7.69)	0.105*** (7.59)	0.012 (1.11)
LEV	−0.110** (−2.49)	−0.101** (−2.19)	0.160*** (3.22)
ROA	0.668*** (6.91)	0.673*** (6.62)	0.765*** (6.38)
TAR	0.010*** (7.11)	0.011*** (15.61)	0.014*** (19.32)
SHARE	0.119* (1.93)	0.123 (1.55)	0.210*** (3.33)
GDP	0.011 (0.47)	0.078 (1.04)	0.099* (1.69)
FDI	−0.042*** (−3.10)	−0.011 (−0.66)	−0.016 (−0.90)
Constant term	−0.380 (−1.52)	−1.493* (−1.68)	−0.522 (−0.81)
Sample size	17,416	17,416	17,416
R2	0.310	0.310	0.262
Id effect	No	No	No
Year effect	Yes	Yes	Yes
Industry effect	Yes	No	Yes
Provincial effect	No	Yes	Yes

#### 4.2.4 Mitigating endogeneity issues

Drawing on Zhang et al. (17), this research employs the air circulation coefficient as an instrumental variable (IV) for green credit. For one thing, the local air circulation coefficient is closely linked to the local environment. This means that when the local air circulation coefficient is low, it makes it harder for pollutants like smog to spread. This means that reducing regional pollution will be given more attention, and stricter environmental protection policies like green credit will be put in place. To meet the requirements of instrumental variables, the chosen air circulation coefficient has a relationship with green credit. Another thing is that the air circulation coefficient is only affected by natural objective factors, and it has enough externalities to meet the needs for instrumental variables. So, the air circulation coefficient of lag phase one is used as the green credit instrument variable in this study. The European Centre for Medium-range Weather Forecasts’ data on atmospheric boundary height and wind speed is used to figure out the regional air circulation coefficient. The study uses enterprise-level robust standard errors and the generalized method of moments to deal with heteroscedasticity. Table 5 presents the empirical findings: Columns (1) and (2) display the first-stage and second-stage results, respectively, using the air circulation coefficient IV. The Kleibergen-Paap rk LM statistic is 22.05 and the *p*-value is 0.00. This shows that there is a strong instrument relevance between the IV and endogenous variables, and the assumption that they are “not identifiable” is not true. The Cragg-Donald F statistic of 25.24 is greater than the 10% threshold level of 16.38, with no evidence of a weak instrumental variable issue. After using the instrumental variable method, the green credit coefficient is still significantly positive. This study adds Green Credit Lag Phase I as a second instrumental variable and uses the enterprise-level clustering robust standard error and GMM method to estimate the tool variable method. This is done to make sure the method is robust. Column (3) reports the first-stage estimates, while Column (4) displays the second-stage outcomes. As shown in the statistics, the Kleibergen-Paap rk LM statistic is 331.08, the *p* value is 0.00, and the Cragg-Donald F statistic 1744.47 is higher than the critical value level of 10% (16.38), which means that the IV and endogenous variables are strongly related to each other and there is no issue of non-identification. It’s important to note that the green credit coefficient stays positive at the 1% level across both IV specifications, which shows that the results are consistent.

**Table 5:** Instrumental variable method.

Variable	Air circulation coefficient		Green credit lags one stage	
	(1)	(2)	(3)	(4)
	GCRE	GTFP	GCRE	GTFP
GCRE		3.000** (2.15)		0.446*** (2.99)
L.FLOW	0.015*** (5.02)			
L.GCRE			0.657*** (41.77)	
SIZE	0.003 (1.20)	0.130*** (6.63)	0.001 (0.95)	0.137*** (7.37)
LEV	0.010 (1.15)	−0.180*** (−2.94)	0.004 (1.05)	−0.156*** (−2.71)
ROA	0.014 (1.07)	0.603*** (6.84)	0.017** (2.55)	0.638*** (7.87)
TAR	0.000 (0.57)	0.009*** (8.96)	0.000 (0.07)	0.009*** (9.23)
SHARE	−0.027* (−1.89)	0.159* (1.78)	−0.009 (−1.43)	0.089 (1.10)
GDP	0.010 (1.02)	0.083 (1.44)	−0.022*** (−4.53)	0.092* (1.77)
FDI	−0.001 (−0.16)	−0.017 (−0.97)	0.003* (1.92)	−0.016 (−1.13)
Sample size	16,172	16,172	16,172	16,172
R2	-	0.105	-	0.301
Id effect	YES	YES	YES	YES
Year effect	YES	YES	YES	YES

### 4.3 Impact mechanism analysis

Building on the above theoretical frameworks and research assumptions, green credit enhances corporate GTFP through pathways like R&D innovation and public oversight. Following Cheng and Kong (21), this research employs a model (2) to examine the relevant mechanism as specified in Equation 2:

(2) $$M_{\mathrm{it}}=\gamma_{0}+\gamma_{1}GCRE_{\mathrm{it}}+\beta X_{\mathrm{it}}+\lambda_{i}+\mu_{t}+\in_{\mathrm{it}}$$

where $M_{\mathrm{it}}$ represents a mechanism variable of firm i’s in the t period, and other variables are defined similarly as in Equation 1.

#### 4.3.1 R&D innovation

Green credit steers enterprises toward green innovation and incentivizes greater investment in sustainable initiatives. Drawing on Cheng and Kong (21) and others, this research employs enterprise research and development investment (INN) and green patent applications (PAT) to assess enterprises’ green innovation propensity. We further examine how green credit enhances GTFP through the R&D innovation mechanism. INN is the amount of research and development investment for the enterprise, sourced from the CSMAR database. The variables undergo standardization to guarantee a uniform magnitude. We quantify corporate green patent applications, with raw data sourced from the CNRDS database. Table 6 systematically presents the empirical findings. Columns (1) and (2) examine the effects of green credit on corporate R&D investment; columns (3) and (4) investigate its influence on the number of green patent applications. The baseline models in Columns (1) and (3) report preliminary estimates without incorporating control variables. In contrast, Columns (2) and (4) incorporate micro-level and macro-level control variables. Green credit exhibits a significantly positive coefficient at a 10% confidence level, suggesting it incentivizes enterprises to boost R&D investment and green patent filings. Improving research and development in businesses can also help them control pollution, make better use of resources, get rid of old production methods, and promote energy conservation, emission reduction, and green transformation (13, 22). This can lead to an increase in the company’s gross foreign product (GFP), which supports the validity of hypothesis 2a.

**Table 6:** R&D innovation mechanism.

Variable	(1)	(2)	(3)	(4)
	INN	INN	PAT	PAT
GCRE	0.549*** (2.61)	0.551*** (2.77)	0.383* (1.77)	0.380* (1.76)
SIZE		0.280*** (8.46)		0.009 (0.61)
LEV		−0.050 (−0.64)		−0.045 (−0.99)
ROA		0.172 (1.38)		−0.002 (−0.03)
TAR		−0.003*** (−2.81)		−0.001*** (−2.96)
SHARE		−0.137 (−0.81)		−0.016 (−0.23)
GDP		0.093 (0.77)		−0.049 (−0.88)
FDI		−0.039 (−1.28)		0.034 (1.33)
Constant term	−0.671*** (−5.46)	−4.746*** (−3.55)	−0.253** (−2.15)	−0.048 (−0.11)
Sample size	10,563	10,563	17,262	17,262
R2	0.177	0.228	0.010	0.010
Id effect	Yes	Yes	Yes	Yes
Year effect	Yes	Yes	Yes	Yes

#### 4.3.2 Public supervision

Green credit policies must be implemented effectively, which means that loan balances and project details must be made public, and the public must be more involved in environmental and green finance issues. To find out how involved the public is in protecting the environment and how well corporations are doing at doing their part, this study looks at the number of proposals on the environment (PSV) and the number of proposals on the environment (PPO) of the local CPPCC. The research also examines the mediating role of public accountability mechanisms in enhancing corporate GTFP through green credit. PSV and PPO quantify the number of environmental proposals submitted by regional committees of the Chinese People’s Political Consultative Conference, with data derived from the China Environmental Yearbook. As shown in Table 7, empirical results systematically evaluate this pathway. The first two columns look at how green credit affects the number of environmental proposals made by the regional CPPCC. The third and fourth columns show the regression results of green credit on the number of environmental proposals made by the regional CPPCC. Columns (1) and (3) report preliminary estimates excluding control variables, whereas columns (2) and (4) are augmented with micro and macro controls. Results consistently show a significantly positive coefficient for green credit, demonstrating its capacity to amplify the number of proposals on environmental issues and increase public attention to and supervision of environmental protection. Businesses cannot avoid their environmental duties if public oversight gets better. Businesses will have to take steps to improve their processes, spend less on polluting projects, and cut down on pollution from production. This will lead to a greener business environment (25) and higher green total factor productivity. These verified linkages support Hypothesis 2a.

**Table 7:** Public oversight mechanism.

Variable	(1)	(2)	(3)	(4)
	PSV	PSV	PPO	PPO
GCRE	0.669*** (6.83)	0.587*** (5.59)	0.317*** (3.63)	0.191** (1.97)
SIZE		−0.012 (−0.82)		−0.011 (−0.81)
LEV		−0.026 (−0.45)		−0.021 (−0.39)
ROA		0.223* (1.86)		0.155 (−0.39)
TAR		−0.000 (−1.52)		−0.000 (−1.13)
SHARE		0.129 (1.35)		0.135 (1.57)
GDP		0.184*** (3.82)		0.200*** (3.44)
FDI		0.186*** (12.68)		0.260 (13.03)
Constant term	−0.460*** (−9.08)	−3.462*** (−6.83)	−0.355*** (−7.79)	−3.995*** (−6.68)
Sample size	14,928	14,928	14,928	14,928
R2	0.087	0.090	0.153	0.159
Id effect	Yes	Yes	Yes	Yes
Year effect	Yes	Yes	Yes	Yes

### 4.4 Constraints on financing

According to the research of Hadlock and Pierce (23), the SA index (SA) and KZ index (KZ) were used to measure corporate financing constraints, respectively. Among them, SA represents the enterprise SA index, KZ represents the enterprise KZ index, and the larger the SA or KZ, the more severe the financing constraints faced by the enterprise. The data is sourced from the CSMAR database. Columns (1) and (2) of Table 8 show the regression results of SA index on green credit, while the third and fourth columns show the regression results of KZ index on green credit. The first and third columns do not include control variables, while the second and fourth columns include micro - and macro level control variables. The results showed that after adding control variables, the regression coefficient of green credit was significantly positive at the 1% confidence level, indicating that green credit can significantly improve corporate financing constraints. The improvement of financing constraints can optimize the allocation and utilization efficiency of enterprise funds, guide enterprises to take on more environmental responsibilities.

**Table 8:** Financing constraint mechanism.

Variable	(1)	(2)	(3)	(4)
	SA	SA	KZ	KZ
GCRE	0.066*** (2.70)	0.066*** (2.77)	0.658** (2.40)	0.504*** (2.99)
SIZE		−0.018*** (−2.73)		−0.494*** (−15.11)
LEV		0.085*** (5.69)		5.461*** (44.68)
ROA		−0.048** (−2.36)		−5.077*** (−20.59)
TAR		−0.000 (−1.24)		−0.001 (−0.79)
SHARE		0.040* (1.77)		−0.082 (−0.52)
GDP		−0.008 (−0.58)		0.317*** (3.05)
FDI		−0.002 (−0.48)		−0.029 (−0.82)
Constant term	−3.615*** (−294.96)	−3.362*** (−21.57)	2.017*** (15.39)	2.607** (2.31)
Sample size	17,416	17,416	16,960	16,960
R2	0.811	0.818	0.218	0.537
Id effect	Yes	Yes	Yes	Yes
Year effect	Yes	Yes	Yes	Yes

### 4.5 Heterogeneity analysis

#### 4.5.1 Corporate lifecycle stages

According to the corporate life cycle theory, firms usually experience four cycles in the process of development: start-up, growth, maturity, and decline. Businesses have different strategic priorities, technological abilities, and financial strengths at different stages of their life cycles. As a result, green credit policies have different effects depending on these changing factors (17). Thus, the enterprise life cycle is segmented to assess how green credit impacts firms across developmental stages. Referring to the research of Cheng and Kong (21) and others (24), listed companies usually have passed the start-up period, further dividing the enterprises into three groups—growing, mature, and declining—based on their cash flow. After classifying the companies, the study performs grouped regression analysis, with results shown in columns (1) to (3) of Table 9. The regression results reveal that green credit has a significantly positive effect on the GTFP of growing and mature companies. According to reality, most of the growing enterprises are in emerging industries. They usually have outstanding development potential and strong innovation ability and can promote green innovation in a timely manner. In addition, the mature enterprises have strong profitability and financing ability and can invest in some long-term and high-risk green environmental protection projects. Therefore, green credit has a more pronounced impact on these two types of enterprises. In contrast, the regression results for declining companies, presented in column (3) of Table 8, show that the coefficient of green credit is not significant. This suggests that enterprises in recession usually have poor innovation ability and development potential, i.e., green credit applied to enterprises in recession cannot improve their GTFP.

**Table 9:** Heterogeneous Regression Results.

Variable	(1)	(2)	(3)	(4)	(5)	(6)	(7)	(8)
	Growth stage	Mature period	Recession	Low level of digital transformation	High degree of digital transformation	Non-polluting industry	Medium and low pollution industries	Heavy pollution industry
	GTFP	GTFP	GTFP	GTFP	GTFP	GTFP	GTFP	GTFP
GCRE	0.313*** (2.80)	0.299*** (3.05)	0.329 (1.33)	0.198 (1.27)	0.344*** (3.17)	0.513*** (2.62)	0.246 (1.36)	0.249* (1.88)
SIZE	0.126*** (5.68)	0.155*** (6.98)	0.136*** (3.38)	0.139*** (5.12)	0.161*** (5.65)	0.144*** (4.60)	0.155*** (4.90)	0.141*** (4.50)
LEV	−0.108* (−1.82)	−0.155** (−2.37)	−0.322*** (−2.63)	−0.206** (−2.41)	−0.094 (−1.62)	−0.149 (−1.55)	−0.214*** (−2.75)	−0.157 (−1.28)
ROA	0.773*** (6.19)	0.545*** (4.73)	0.679*** (3.61)	0.884*** (7.11)	0.327*** (3.25)	0.620*** (3.98)	0.642*** (6.54)	0.660*** (4.42)
TAR	0.009*** (6.16)	0.011*** (7.65)	0.009*** (6.64)	0.008*** (5.83)	0.010*** (8.09)	0.008*** (7.14)	0.013*** (4.64)	0.013*** (6.59)
SHARE	0.133 (1.42)	0.136 (1.59)	−0.042 (−0.20)	0.233** (2.50)	0.020 (0.18)	0.025 (0.14)	0.034 (0.24)	0.109 (1.06)
GDP	0.114* (1.90)	0.067 (1.33)	0.113 (0.77)	0.045 (0.56)	0.140** (2.11)	0.099 (0.89)	0.057 (0.90)	0.097 (1.36)
FDI	−0.02 (−1.08)	0.002 (0.12)	−0.016 (−0.55)	−0.018 (−0.77)	−0.023 (−1.44)	−0.052* (−1.74)	−0.014 (−0.67)	0.047*** (2.61)
Constant term	−1.912*** (−2.98)	−1.887*** (−3.06)	−1.896 (−1.22)	−1.313 (−1.59)	−1.690*** (−3.17)	−1.794 (−1.43)	−1.561** (−2.37)	−2.276*** (−2.95)
Sample size	6,970	7,140	3,306	8,386	8,386	5,964	3,906	5,390
R2	0.289	0.337	0.289	0.242	0.334	0.323	0.365	0.341
Id effect	Yes	Yes	Yes	Yes	Yes	Yes	Yes	Yes
Year effect	Yes	Yes	Yes	Yes	Yes	Yes	Yes	Yes

#### 4.5.2 Digital transformation degrees

Businesses with a higher level of digitalization typically focus more on pertinent government policies and are more susceptible to the influence of green credit policies. This makes them more likely to undergo green transformation and enhance their GTFP (17). Therefore, in line with the methodology employed by Cheng and Kong (21) and others, this research adopts the number of words related to digitalization in a company’s annual report to measure the level of digitalization within enterprises. The data, sourced from the CSMAR database, includes keywords related to digitalization, mainly the segmented words of big data technology, application of digital technology, artificial intelligence, cloud computing, and blockchain. Group regression classifies enterprises into low-level and high-level groups based on the median number of digital-related words. The results, shown in columns (4) and (5) of Table 8, reveal distinct impacts of green credit on these groups. For companies with low-level digital transformation, green credit shows no significant effect. However, for enterprises with advanced digital capabilities, green credit demonstrates a significant and positive influence on their GTFP. Enterprises with a high degree of digitalization transformation usually have a strong technological innovation ability and enthusiasm, driving green development through technological advancements. Their high digitalization level also indicates that these enterprises have substantial development potential, can adapt to China’s economic development and industrial structure adjustment, and play a pivotal role in driving corporate green transformation. Green credit policies significantly influence these enterprises. On the other hand, enterprises with a low degree of digital transformation typically lack the ability or willingness to innovate, can only respond passively to the pressures imposed by green credit initiatives, and are unable to timely enhance their GTFP.

#### 4.5.3 Industry pollution levels

Green credit has a markedly different impact on polluting and non-polluting enterprises. This divergence arises because companies with different levels of environmental impact adopt distinct strategies when responding to green credit policies (17). Based on the level of pollution in an industry, this study uses a classification method like that of Cheng and Kong (21) to investigate how green credit affects the GTFP of businesses in different ways. We categorize the businesses into four groups: heavy pollution, medium and low pollution, and non-pollution industries. Columns (6)–(8) in Table 8 show the empirical analysis, which shows that green credit has different effects at different levels of pollution. The findings indicate that green credit substantially enhances the GTFP of both clean and heavily polluting enterprises. However, for enterprises in medium- and low-pollution industries, the influence of such financial instruments is not statistically significant. Environmental concerns must be considered by financial institutions when approving green credit. Polluting projects must be limited, and credit resources must be made available for businesses’ green projects. This encourages businesses that do not pollute to pursue green production and innovation activities, which increases their GTFP. At the same time, heavily polluting enterprises, which are a primary focus of such policies, face increased scrutiny and pressure to adopt sustainable practices, driving them toward greener operations. The implementation of stricter financial regulations forces heavily polluting enterprises to adopt measures to reduce their environmental impact. This pressure drives them to invest in cleaner technologies and sustainable practices, facilitating their green transformation. As a result, green credit emerges as a key factor in enhancing the GTFP of these enterprises. In addition, for enterprises in medium- and low-pollution industries, green credit has insufficient power to drive significant improvements in GTFP. While the correlation coefficient between green credit and these enterprises exhibits a positive trend, it fails to achieve statistical significance.

## 5 Concluding remarks and policy implications

The research examines the influence of green credit on corporate GTFP using panel data analysis of A-share listed entities on the Shanghai and Shenzhen stock exchanges between 2008 and 2021. The empirical outcomes can be categorized into three principal dimensions. Primarily, the findings demonstrate that green credit substantially contributes to the enhancement of corporate GTFP, supporting their green transformation and upgrading. These results maintain their validity across multiple robustness checks, encompassing replacing variables, narrowing the sample period, adjusting fixed effect dimensions, and applying the instrumental variable method. Secondly, mechanism analysis reveals two primary channels through which green credit promotes sustainable business transformation and enhances their GTFP: stimulating investments in research and innovation and strengthening public oversight. Thirdly, the heterogeneity analysis shows that green credit demonstrates substantial effectiveness in improving green total factor productivity for organizations with a high degree of growth, maturity, and digital transformation. However, its effect is not statistically significant for companies experiencing decline or those with limited digital capabilities. In addition, green credit has a significant positive effect on GTFP in clean and heavily polluting industries. However, its impact on moderately and minimally polluting industries does not reach statistical significance.

Based on the findings of this research, several policy recommendations can be formulated. First, maximize the effectiveness of eco-friendly credit mechanisms and strengthen the connection between green initiatives and financial systems. Green credit policies should focus on the green transformation of highly polluting enterprises and promote the transformation of enterprises to green production mode by restricting the financing of polluting projects. In the meantime, funds should be directed to specific key areas, and weak links should be supported by the state to effectively prevent factors such as overcapacity, high energy consumption, and pollution from flowing into non-green economic areas. Additionally, it is vital to accelerate research and innovation within enterprises while leveraging public oversight to ensure accountability. Encouraging green innovation is a critical step in enhancing GTFP, as it helps to both enhance environmental conditions and cut production costs. Under the current background of optimizing credit structure, green development, and innovating monetary policy tools, the public can be made aware through information disclosure, mobilize public enthusiasm for environmental protection, enhance external oversight of corporate practices by the public, broaden public participation channels, and strengthen the publicity and guidance of policy implementation details. Finally, it is crucial to enhance the information supervision system and strengthen the management of environmental protection information disclosure. Focus on making it easier for environmental data to be shared quickly and easily by combining policies that support financial technology and digital transformation. This approach will help reduce information asymmetry between stakeholders and polluting enterprises, fostering greater transparency and accountability in environmental practices.

## Data Availability

The original contributions presented in the study are included in the article/supplementary material, further inquiries can be directed to the corresponding author.
